# Curcumin, a Multi-Ion Channel Blocker That Preferentially Blocks Late Na^+^ Current and Prevents I/R-Induced Arrhythmias

**DOI:** 10.3389/fphys.2020.00978

**Published:** 2020-08-21

**Authors:** Lv Song, Ze-fu Zhang, Liang-kun Hu, Pei-hua Zhang, Zhen-zhen Cao, Zhi-pei Liu, Pei-pei Zhang, Ji-hua Ma

**Affiliations:** ^1^Cardio-Electrophysiological Research Laboratory, Medical College of Wuhan University of Science and Technology, Wuhan, China; ^2^College of Life Science and Health, Wuhan University of Science and Technology, Wuhan, China; ^3^Hubei Province Key Laboratory of Occupational Hazard Identification and Control, Medical College of Wuhan University of Science and Technology, Wuhan, China; ^4^Tianyou Hospital Affiliated to Wuhan University of Science and Technology, Wuhan, China

**Keywords:** curcumin, ionic currents, action potential, ventricular myocytes, arrhythmias, ischemia–reperfusion

## Abstract

Increasing evidence shows that Curcumin (Cur) has a protective effect against cardiovascular diseases. However, the role of Cur in the electrophysiology of cardiomyocytes is currently not entirely understood. Therefore, the present study was conducted to investigate the effects of Cur on the action potential and transmembrane ion currents in rabbit ventricular myocytes to explore its antiarrhythmic property. The whole-cell patch clamp was used to record the action potential and ion currents, while the multichannel acquisition and analysis system was used to synchronously record the electrocardiogram and monophasic action potential. The results showed that 30 μmol/L Cur shortened the 50 and 90% repolarization of action potential by 17 and 7%, respectively. In addition, Cur concentration dependently inhibited the Late-sodium current (*I*_Na.L_), Transient-sodium current (*I*_Na.T_), L-type calcium current (*I*_Ca.L_), and Rapidly delayed rectifying potassium current (*I*_Kr_), with IC_50_ values of 7.53, 398.88, 16.66, and 9.96 μmol/L, respectively. Importantly, the inhibitory effect of Cur on *I*_Na.L_ was 52.97-fold higher than that of *I*_Na.T_. Moreover, Cur decreased ATX II-prolonged APD, suppressed the ATX II-induced early afterdepolarization (EAD) and Ca^2+^-induced delayed afterdepolarization (DAD) in ventricular myocytes, and reduced the occurrence and average duration of ventricular tachycardias and ventricular fibrillations induced by ischemia–reperfusion injury. In conclusion, Cur inhibited *I*_Na.L_, *I*_Na.T_, *I*_Ca.L_, and *I*_Kr_; shortened APD; significantly suppressed EAD and DAD-like arrhythmogenic activities at the cellular level; and exhibited antiarrhythmic effect at the organ level. It is first revealed that Cur is a multi-ion channel blocker that preferentially blocks *I*_Na.L_ and may have potential antiarrhythmic property.

## Introduction

Cardiovascular diseases (CVDs) are the leading global cause of mortality, and approximately half of this mortality contributes to sudden cardiac deaths (SCDs) (Go et al., 2014), which generally occur after malignant arrhythmia. Most traditional antiarrhythmic drugs (AADs) are selective ion channel blockers, a large part of which are limited in clinical application due to their potential proarrhythmic effects and numerous side effects (Zdanowicz and Lynch, 2011). However, the commonly used AAD (amiodarone) is a potent hERG channel inhibitor with a lower risk of proarrhythmic effect because of a counteraction to K^+^ current inhibition by either Na^+^ or Ca^2+^ channel blockade (Kodama et al., 1997; Martin et al., 2004). At present, it is generally believed that an ideal AAD should have multi-ion channel effects, and one of which is the best target (Budillon et al., 2005; Nattel and Carlsson, 2006; Barman, 2015; Polak et al., 2015). Modern cardiology is still waiting for the introduction of safe and effective AADs. Over the years, cumulative randomized controlled trials of traditional natural products have demonstrated its efficacy and safety in patients with CVDs and gradually popularized worldwide (Brenyo and Aktas, 2014; Wang et al., 2014; Hua et al., 2019).

**FIGURE 1:**
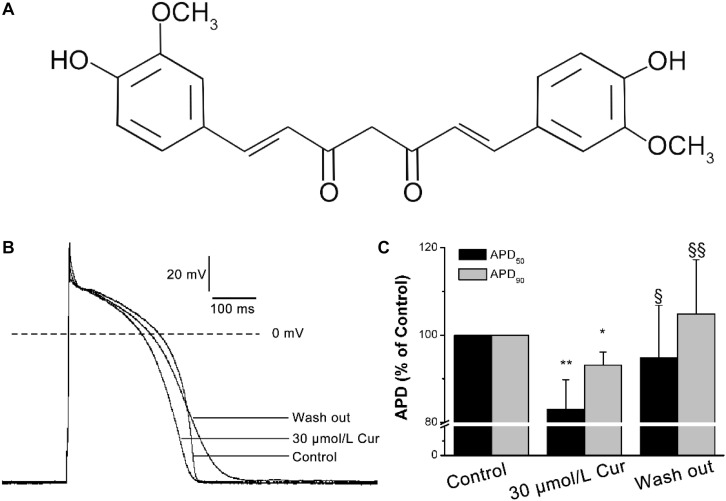
Effects of Curcumin (Cur) on action potentials (APs) in ventricular myocytes. **(A)** Structural formula of Cur. **(B)** Example records of APs from the control group, Cur group, and wash out group in the same ventricular myocyte. **(C)** The comparisons of the average APD_50_ and APD_90_ percentage values of the control group, Cur group, and wash out group (*n* = 12, *P < 0.05, **P < 0.01 vs. control; ^§^ *P* < 0.05, ^§§^ *P* < 0.01 vs. 30 μmol/L Cur).

Curcumin (Cur: C_2__1_H_2__0_O_6_, MW:368.38, Figure 1A) is a natural yellow polyphenolic substance, the main active alkaloid extracted from the rhizome of turmeric, a rhizomatous herbaceous perennial plant belonging to the family Zingiberaceae, which has been used as an antiseptic and antipyretic folk medicine for centuries (Kumar et al., 2017). Previous researches have shown that Cur has extensive pharmacological activities (Aggarwal et al., 2007; Yallapu et al., 2015; Salehi et al., 2019; Salehi et al., 2020a, b) and has been put into clinical practice with a dose range of 180–3000 mg/day (Panahi et al., 2014). Increasing evidence showed that Cur has a protective effect against CVDs. For instance, Cur can prevent the development of heart failure by inhibiting p300 histone acetyltransferase activity (Morimoto et al., 2008), antagonized sodium fluoride intoxication in rat heart (Nabavi et al., 2011), prevented isoprenaline (ISO)-induced cardiac hypertrophy (Izem-Meziane et al., 2012), and can have a protective effect against the myocardial infarction injury (Nirmala and Puvanakrishnan, 1996). In addition, Cur was reported to prevent the QTc prolongation in ISO-induced myocardial infarction (Boarescu et al., 2019). However, Hu et al. found that Cur blocked hERG channel but had no significant effect on QT interval of rabbits (Hu et al., 2012). At present, few researches have been done on the effects of Cur on cardiac electrophysiology. Therefore, we carry out this study to better understand the electrophysiological properties of Cur and explore its antiarrhythmic property.

## Materials and Methods

### Ventricular Myocytes Preparation

All animal experiments in this investigation were performed in accordance with the *Guide for the Care and Use of Laboratory Animals* (NIH publication No. 85-23, revised 1996) and approved by the Institutional Animal Care and Use of Wuhan University of Science and Technology. Healthy adult New Zealand white rabbits of either sex, weighing 1.8–2.2 kg, were anesthetized with xylazine (Shanghai Shifeng Biological Co., Ltd., Shanghai, China, 7.5 mg/kg i.m.) and ketamine (Fujian Gutian Pharmaceutical Co., Ltd., Fujian, China, 30 mg/kg i.v.); after heparinization, thoracotomy was performed to quickly remove the heart, which was then placed in oxygen-saturated Ca^2+^-free Tyrode solution containing the following (in mM): 135 NaCl, 5.4 KCl, 1 MgCl_2_, 0.33 NaH_2_PO_4_, 10 HEPES, and 10 glucose (titrated to pH 7.4 with NaOH). The cardiac aorta was retrogradely cannulated and hanged on the Langendorff-perfusion device, followed by perfusing with Ca^2+^-free Tyrode solution for 5 min, and then perfused with Ca^2+^-free Tyrode solution containing collagenase (Sigma-Aldrich Co, St. Louis, MO, United States, type I, 1 mg/ml) and BSA (Roche Co., Basel, Switzerland, 1 mg/ml) for 40 min. Before isolating the left ventricle, the heart was perfused with KB solution containing the following (in mM): 85 KOH, 30 KCl, 15 KH_2_PO_4_, 6 MgSO_4_, 20 taurine, 10 glucose, 0.5 EGTA, 10 HEPES, and 50 L-glutamic acid (titrated to pH 7.4 with KOH) with BSA (1 mg/ml) for 5 min to terminate the enzymatic hydrolysis. The isolated left ventricle was snipped into small chunks and placed in KB solution containing BSA (1 mg/ml), gently blown to disperse the cells, and finally filtered through a nylon mesh. Individual ventricular myocytes were stored at 4°C in the same solution for 1 h before electrophysiological testing. All perfusates were saturated with O_2_ and maintained at 37 ± 0.5C with a thermostatic bath.

### Whole-Cell Patch-Clamp Preparation

Whole-cell patch-clamp recording was performed on single ventricular myocyte using an EPC10 amplifier (HEKA Electronic, Lambrecht, Germany), filtered at 2 kHz, and digitized at 10 kHz. Electrodes were fabricated from borosilicate glass using a two-stage microelectrode puller (PP-100, Narishige Group, Tokyo, Japan) and tip resistances of 1.5–2.5 MΩ when filled with pipette solution. The cell suspension was placed in a 1 ml chamber mounted on the stage of a microscope (TE2000; Nikon, Japan) for about 5 min and then perfused with external solution at a rate of 2 ml/min via an FR-50 solution flow control valve (Warner Instruments Inc., United States) after the cells were completely attached to the chamber wall. The single ventricular myocyte with stable adherence, clear striations, and high refractive index was selected for recording. The electrode filled with pipette solution was inserted into the external solution using 3-D micro-manipulation (MP285, Sutter, United States). The Giga-Ohm seal was formed by the negative pressure, and then the whole-cell clamp mode with a series resistance of 2–5 MΩ was obtained by rupturing the cell membrane. Slow capacitance was routinely optimized and series resistance was compensated by 60–80%. All external solutions were saturated with O_2_ and experiments were conducted at room temperature (22–25°C).

### APs and Ionic Currents Recordings

Ionic currents were recorded in a whole-cell mode of voltage-clamp configuration and the APs were recorded in a current-clamp configuration after the Giga-Ohm seal is implemented in voltage-clamp mode. Voltage protocols, pipette solutions, and external solutions were designed to record different ionic currents.

The pipette solution of APs contained the following (in mM): 5 NaCl, 30 KCl, 110 K-aspartate, 5 Mg-ATP, 1 EGTA, 10 HEPES, 5 creatine phosphate, and 0.5 CAMP (titrated to pH 7.3 with KOH). The external solution of APs contained the following (in mM): 145 NaCl, 5.6 KCl, 1.8 CaCl_2_, 1.2 MgCl_2_, 5 HEPES, and 10 glucose (titrated to pH 7.3 with NaOH).

The pipette solution of *I*_Na.L_ contained the following (in mM): 120 CsCl, 1 CaCl_2_, 5 MgCl_2_, 5 Na_2_ATP, 10 tetraethylammonium chloride (TEA-Cl), 11 EGTA, and 10 HEPES (titrated to pH 7.3 with CsOH). There was no difference between the pipette solution of *I*_Na.L_, *I*_Ca.L_, and *I*_Na.T_. The external solution of *I*_Na.L_ contained the following (in mM): 135 NaCl, 5.4 CsCl, 1 MgCl_2_, 10 glucose, 0.33 NaH_2_PO_4_, 0.3 BaCl_2_, 10 HEPES, and 1.8 CaCl_2_ (titrated to pH 7.4 with NaOH). The composition of external solution used to record *I*_Ca.L_ and *I*_Na.L_ was the same. However, 10 μmol/L nifedipine were added to the external solution to block *I*_Ca.L_ when recording *I*_Na.L_. The low-sodium external solution was designed to record *I*_Na.T_, which contain the following (in mM): 30 NaCl, 1 CaCl_2_, 105 CsCl, 1 MgCl_2_, 0.05 CdCl_2_, 5 HEPES, and 10 glucose (titrated to pH 7.4 with CsOH).

The pipette solution of *I*_Kr_ contained the following (in mM): 140 KCl, 5 K_2_ATP, 1 MgCl_2_, 10 EGTA, and 5 HEPES (titrated to pH 7.3 with KOH). The external solution contained the following (in mM): 135 NaCl, 1 MgCl_2_, 5.4 KCl, 0.33 NaH_2_PO_4_, and 10 HEPES (titrated to pH 7.4 with NaOH). In order to eliminate the interference of the other currents, 10 μmol/L nifedipine (*I*_Ca.L_ blocker), 0.2 mmol/LBaCl_2_ (I_K1_ blocker), 0.2 mmol/L 4-AP (I_to_ blocker), and 30 μmol/L 293B (I_Ks_ blocker) were added to the external solution when recording I_kr_.

CsCl and KH_2_PO_4_ were purchased from Amresco (Solon, OH, United States), while EGTA and HEPES were purchased from Biosharp (Hefei, China). The other unspecified drugs were bought from Sigma-Aldrich (St. Louis, MO, United States).

For the AP recordings, we need to switch whole-cell clamp mode to current-clamp mode. APs were elicited by continuously stimulating the cells with suprathreshold (1.2–1.5 times) depolarizing pulses (6 ms duration) at a frequency of 1 Hz delivered via the patch pipette.

For the *I*_Ca.L_ recordings, the holding potential (HP) was −40 mV. The single *I*_Ca.L_ was elicited by a depolarizing pulse to 0 mV for 300 ms from the HP. The current–voltage (*I*–*V*) relationships of *I*_Ca.L_ was obtained by the 300-ms depolarizing steps from −40 to +60 mV in 5 mV increments. The steady-state inactivation of *I*_Ca.L_ was elicited by the use of a two-pulse protocol: stepping from −50 to 0 mV in 5 mV increments for 2 s followed by a 300 ms depolarizing pulse to 0 mV at 2 s intervals. The time-dependent recovery of *I*_Ca.L_ was determined by two 300 ms depolarizing pulses to 0 mV from the HP, the former of which is a condition pulse and the latter is a test pulse with intervals from 0 to 3100 ms in 100 ms increments.

For the *I*_Na.T_ and *I*_Na.L_ recordings, the HP was −90 mV. The single *I*_Na.T_ was elicited by a depolarizing pulse to −30 mV from HP, and the single *I*_Na.L_ was elicited by a depolarizing pulse to −20 mV and measured at 200 ms. The *I*–*V* relationships of *I*_Na.L_ were obtained by applying 300 ms depolarizing steps from −80 to +60 mV in 10 mV increments. Interval between the depolarizing steps was 2 s.

For the *I*_Kr_ recordings, the HP was −40 mV. The *I*–*V* relationships of Cur on *I*_Kr_ was elicited by 3000 ms depolarizing steps from −40 to +50 mV in 10 mV increments, and each depolarizing step is followed by a repolarizing pulse to −40 mV for 5 s to evoke large, slowly decaying outward tail currents (*I*_K__–__tail_).

### Electrocardiogram (ECG) and Monophasic Action Potential (MAP) Recordings of Langendorff-Perfused Isolated Hearts

The antiarrhythmic property of Cur was determined by 30 μmol/L Cur on arrhythmias induced by ischemia-reperfusion (I/R) injury. ECG was recorded by three platinum electrodes, which had been placed near the cardiac apex, right atrium, and aortic root, while MAP was recorded by an electrode placed on the surface of the ventricle. ECG and MAP were monitored simultaneously for 150 min with a multichannel acquisition and analysis system (BL-420F, Taimeng Technology Instruments, Chengdu, china). In the I/R group, within 30 min, the hearts were perfused with Tyrode solution to determine whether they should be considered in the subsequent research; a heartbeat with a disordered rate or rhythm is an exclusion criterion. The arrhythmias were induced by stopping the perfusion for 60 min and then reperfusion for 60 min. In the Cur-I/R group, the perfusate was completely replaced with Tyrode solution containing 30 μmol/L Cur (Sigma-Aldrich Co.) before ischemia to observe the antagonistic effect of Cur on arrhythmias induced by I/R. The Cur used in the whole study was dissolved in DMSO, and the concentration of DMSO in the perfusate did not exceed 1‰.

### Data Analysis and Statistic

Fitmaster (v2x32, HEKA Electronic) and Origin8.0 (Originlab, Northampton, MA, United States) were used for measurement, statistical analysis, and graphic fitting. The concentration–response curves were fitted with a Hill function of *Y* = *B*_max_/[1 + (IC_50_/*D*)*^*n*^*], where *Y* represents percentage inhibition of currents, *n* is the Hill coefficient, *B*_max_ is the maximum inhibitory percentage of currents, and *D* is the Cur concentration. The steady-state (in)activation curves were fitted with a Boltzmann function of *Y* = 1/[1 + exp (*V*_m_ – *V*_1/2_)/*k*], where *Y* represents relative currents (*I*/*I*_max_) of steady-state inactivation and the relative conductance (*G*/*G*_max_) of steady-state activation, respectively. *V*_1/2_ is half (in)activation potential, and *V*_m_ and *k* are the depolarization potential and slope factor, respectively. The conductance (*G*) was calculated by *G* = *I*/(*V*_m_ − *V*_rev_) with the data in the *I*–*V* curves, where *V*_rev_ is the reversal potential. The time-dependent recovery curve was fitted with an exponential function of *Y* = 1 − exp(−*t*/τ), where *Y* represents *I*/*I*_max_ and *t* is the time interval between two pulses. The time constant τ reflects the rate of recovery from inactivation. The current data were normalized (current divided by capacitance) and expressed as current density. Data were presented as means ± SD. Parametric tests included Student’s *t*-test and one-way repeated ANOVA, which were followed by the Bonferroni test for two groups and multiple comparisons, respectively. *P* < 0.05 was considered significant.

## Results

### Effects of Cur on APs

The results showed that there was no significant effect of Cur on resting membrane potential (RMP), action potential amplitude (APA), or maximum depolarization velocity (*V*_max_) (Table 1). Yet, Cur shortened APD_50_ by 17% but only shortened APD_90_ by 7% (Figure 1C). After continuous washing to eliminate the effects of the drug, the APD_50_ and APD_90_ were prolonged (Figure 1B).

**TABLE 1:** Effects of Cur on the parameters of APs in rabbit ventricular myocytes.

**Parameters**	**Control**	**30 μmol/L Cur**	**Wash out**
RMP (mV)	−773	−754	−745
APA (mV)	1237	1149	11410
APD50 (ms)	23915	19922**	22621^§^
APD90 (ms)	2718	25211*	28229^§§^
*V*_max_ (V/s)	18612	16425	15918

**RMP, Resting membrane potential; APA, action potential amplitude; APD_50_, 50% repolarization of AP; APD_90_, 90% repolarization of AP; V_*max*_, maximum depolarization velocity (n = 12, ^∗^P < 0.05, ^∗∗^P < 0.01 vs. control; ^§^P < 0.05, ^§§^P < 0.01 vs. 30 μmol/L Cur).**

### Effects of Cur on *I*_Ca.L_

**FIGURE 2:**
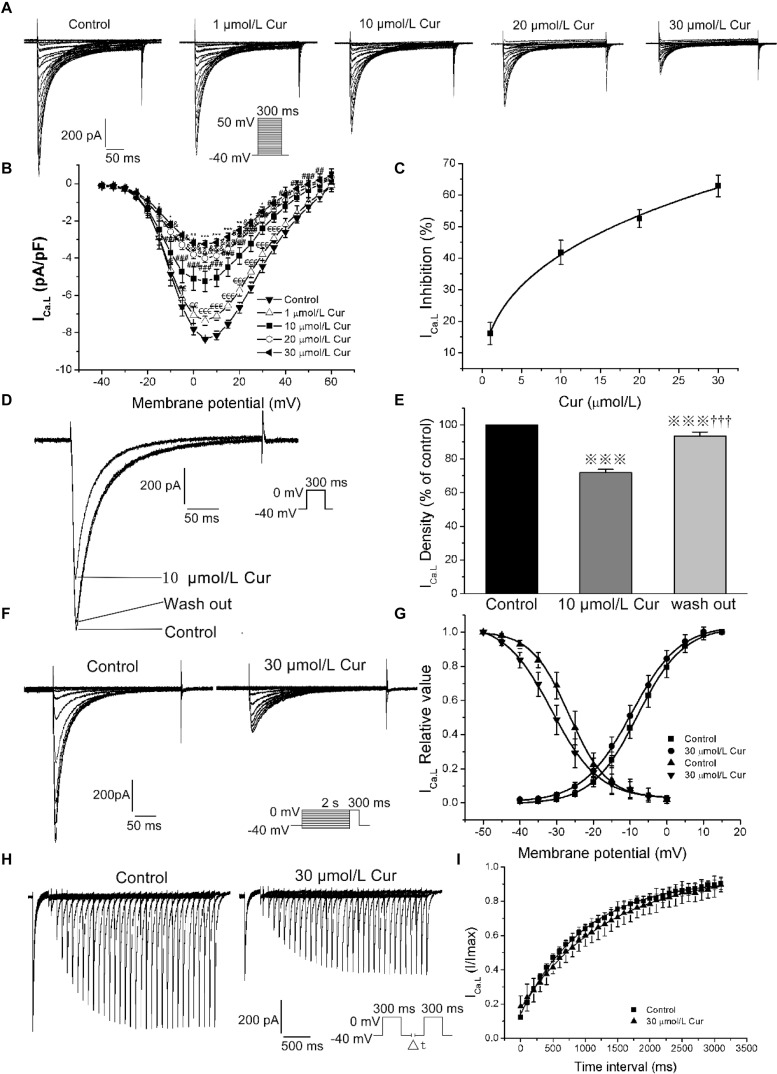
The effects of Cur on L-type calcium current (*I*_Ca.L_) in ventricular myocytes. **(A)** Example records *I*_Ca.L_ in different membrane potentials for the control group and Cur (5, 10, 20, and 30 μmol/L) groups. **(B)** The current–voltage (*I*–*V*) relationship curves of Cur on *I*_Ca.L_ (*n* = 8, ^€^*P* < 0.05, ^€€^*P* < 0.01, ^€€€^*P* < 0.001 vs. control; ^#^*P* < 0.05, ^##^*P* < 0.01, ^###^*P* < 0.001 vs. 5 μmol/L; ^&^*P* < 0.05, ^&&^*P* < 0.01, ^&&&^*P* < 0.001 vs. 10 μmol/L; *P < 0.05, **P < 0.01, ***P < 0.001 vs. 20 μmol/L). **(C)** The concentration–response relationship curve of Cur on *I*_Ca.L_. **(D)** Example records of the single *I*_Ca.L_ from the same cell of three groups (control group, 10 μmol/L Cur group and wash out group), showing a reversible inhibitory effect of Cur on *I*_Ca.L_. **(E)** The comparison of average *I*_Ca.L_ percentage values of the three groups (*n* = 6, 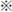
*P* < 0.001 vs. control; ^†††^*P* < 0.001 vs. 10 μmol/L Cur). **(F)** Example records of steady-state inactivation of *I*_Ca.L_ before and after administrating 30 μmol/L Cur. **(G)** Steady-state activation and inactivation curves of two groups. **(H)** Example records of time-dependent recovery of *I*_Ca.L_ before and after 30 μmol/L Cur administration. **(I)** Time-dependent recovery curves of the two groups.

According to our previous literature, the recording of the *I*_Ca.L_ was started 10 min after the whole-cell mode was established and the entire experimental record was finished in 20 min to eliminate the deviation induced by *I*_Ca.L_ rundown (Jiang et al., 2017). By studying the effects of Cur on the *I*–*V* relationships, steady-state (in)activation, and time-dependent recovery of *I*_Ca.L_ in ventricular myocytes, the results showed that Cur inhibited *I*_Ca.L_ in a concentration-dependent manner (Figure 2A) and the maximum current density of *I*_Ca.L_ was obtained at the test potential of 5 mV (Figure 2B). The concentration–response curve fitted with a Hill function with the IC_50_ value of *I*_Ca.L_ was calculated to be 16.66 μmol/L (Figure 2C). The *I*_Ca.L_ was inhibited by 10 μmol/L Cur and reversed by washing out, indicating that the inhibitory effect of Cur on *I*_Ca.L_ was reversible (Figures 2D,E), and 30 μmol/L Cur induced a left shift of steady-state inactivation with the inactivation *V*_1/2_ shifted from −27.17 ± 0.24 to −31.29 ± 0.13 (*n* = 20, *P* < 0.001 vs. control) and *k* was increased from 6.27 ± 0.17 to 7.12 ± 0.27 (*n* = 20, *P* < 0.001 vs. control, Figures 2F,G), with no significant effect on activation (Figure 2G). Cur also slowed the recovery from inactivation with τ value increasing from 988.83 ± 22.49 to 1379.22 ± 29.63 (*n* = 7, *P* < 0.01 vs. control) (Figures 2H,I).

### Effects of Cur on *I*_Na.L_ and *I*_Na.T_

**FIGURE 3:**
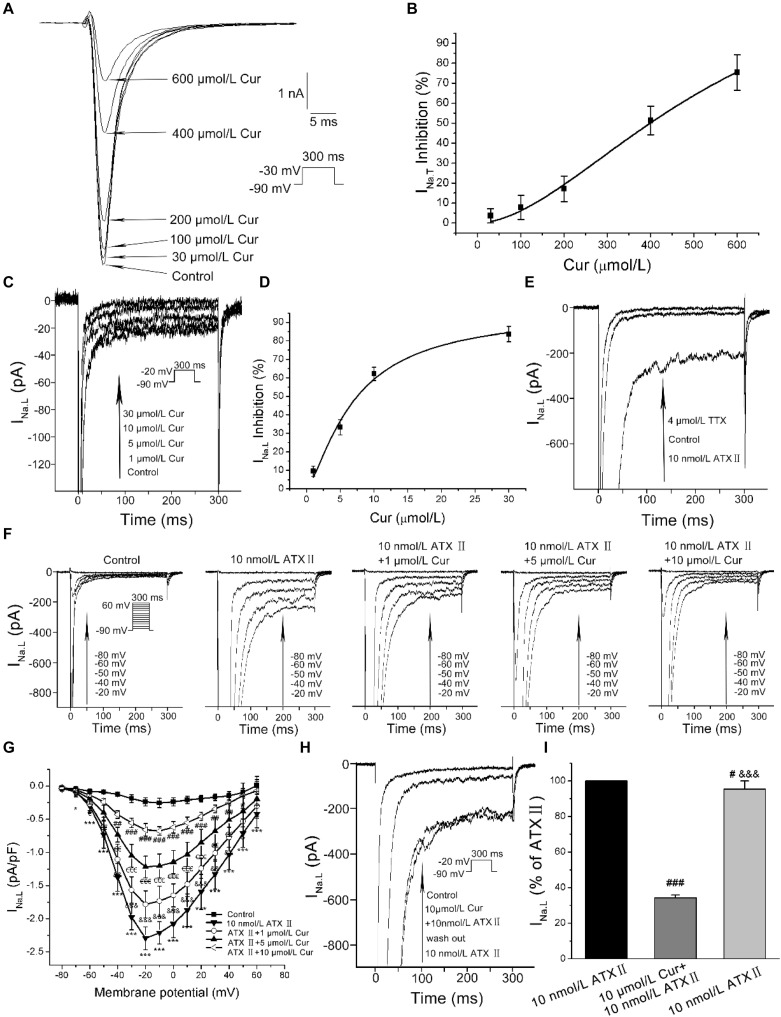
The effects of Cur on transient sodium currents (*I*_Na.T_) and late sodium currents (*I*_Na.L_) in ventricular myocytes. **(A)** Example records of the single *I*_Na.T_ of control group and Cur (30, 100, 200, 400, and 600 μmol/L) groups. **(B)** The concentration–response relationship curve of Cur on *I*_Na.T_. **(C)** Example records of different concentrations of Cur on the basal endogenous *I*_Na.L_. **(D)** The concentration–response relationship curve of Cur on the single basal endogenous *I*_Na.L_. **(E)** Example records of *I*_Na.L_ of control, ATX II, and TTX groups. **(F)** Example records of ATX II increased-*I*_Na.L_ at membrane potentials of −80, −60, −50, −40, and −20 mV from the same cell for the control group, ATX II group, and Cur groups (1, 5, and 10 μmol/L). **(G)** The *I*–*V* relationships curves for the effects of Cur on ATX II-increased *I*_Na.L_ (*n* = 11, *P < 0.05, ***P < 0.001 vs. control; ^&^*P* < 0.05, ^&&^*P* < 0.01, ^&&&^*P* < 0.001 vs. 10 nmol/L ATX II; ^€^*P* < 0.05, ^€€^*P* < 0.01, ^€€€^*P* < 0.001 vs. 1 μmol/L Cur; ^#^*P* < 0.05, ^##^*P* < 0.01, ^###^*P* < 0.001 vs. 5 μmol/L Cur). **(H)** Example records of single *I*_Na.L_ showing a reversible inhibitory effect of Cur on ATX II-increased *I*_Na.L_. **(I)** The comparison of average *I*_Na.L_ percentage values of the three groups (*n* = 8, ^#^*P* < 0.05, ^###^*P* < 0.001 vs. control; ^&&&^*P* < 0.001 vs. 10 μmol/L Cur).

At the given concentration of 30 μmol/L, Cur only reduced the *I*_Na.T_ by 4%, and when the concentration increased to 100, 200, 400, and 600 μmol/L, the *I*_Na.T_ was gradually suppressed more significantly (Figure 3A). The concentration-dependent curve was fitted with a Hill function with the IC_50_ value calculated to be 398.88 μmol/L (Figure 3B). In addition, Cur reduced the basal endogenous *I*_Na.L_ in a concentration-dependent manner with reduced *I*_Na.L_ by 83% at the administration of 30 μmol/L Cur (Figure 3C), and the concentration–response relationship curves were fitted with a Hill function with the IC_50_ value calculated to be 7.53 μmol/L (Figure 3D). The IC_50_ ratio of Cur on *I*_Na.T_ and *I*_Na.L_ was 52.97, which means that the inhibitory effects of Cur on *I*_Na.L_ was 52.97-fold favorable than that of *I*_Na.T_. In the next series of experiments, we used the *I*_Na.L_ opener–Sea anemone toxin II (ATX II, Alomone Labs, Jerusalem, Israel) to induce an increase in the amplitude of *I*_Na.L_ to study the effects of Cur on the enhanced *I*_Na.L_. The amplitude of recorded current was first augmented by 10 nmol/L ATX II, and then sodium channel blocker-TTX (Alomone Labs) at the concentration of 4 μmol/L was applied to the ATXII-increased currents to determine whether the current we recorded was *I*_Na.L_. The results showed that the density of recorded currents was increased from −0.30 ± 0.04 to −1.93 ± 0.14 pA/pF (*n* = 12, *P* < 0.001 vs. ATX II) by 10 nmol/L ATX II, and then reduced to 0.11 ± 0.03 pA/pF (*n* = 12, *P* < 0.001 vs. control) by 4 μmol/L TTX (Figure 3E). It can be concluded that the currents we recorded were *I*_Na.L_. Applying the different concentrations (1, 5, and 10 μmol/L) of Cur on ATXII-increased *I*_Na.L_ (Figure 3F), the *I*–*V* curves showed that Cur effectively inhibited the ATX II-increased *I*_Na.L_ (Figure 3G). In addition, ATX II-increased *I*_Na.L_ was significantly reduced with 10 μmol/L Cur and then increased again after wash out with 10 nmol/L ATX II (Figures 3H,I), which showed that the inhibitory effect of Cur on ATX II-increased *I*_Na.L_ was reversible.

### Effects of Cur on *I*_Kr_

**FIGURE 4:**
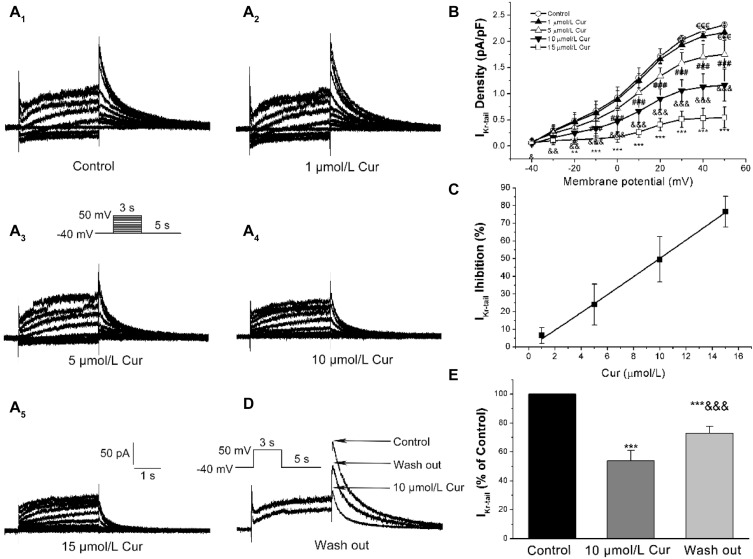
Effects of Cur on rapidly delayed rectifying K^+^ currents (*I*_Kr_) in ventricular myocytes. **(A_1_–A_5_)** Example records of Cur on *I*_Kr_ in different membrane potentials for the control group and Cur groups (1, 5, 10, and 15 μmol/L). **(B)** The *I*–*V* relationships curves for the effects of Cur on *I*_Kr__–__tail_ (*n* = 15, ^€€^*P* < 0.01, ^€€€^*P* < 0.001 vs. control; ^#^*P* < 0.05, ^###^*P* < 0.001 vs. 1 μmol/L Cur; ^&^*P* < 0.05, ^&&^*P* < 0.01, ^&&&^*P* < 0.001 vs. 5 μmol/L Cur; **P < 0.01, ***P < 0.001 vs. 10 μmol/L Cur). **(C)** The concentration–response relationship curve of Cur on *I*_Kr__–__tail_, fitted with a Hill function. **(D)** Example records of single *I*_Kr_ showing a reversible inhibitory effect of Cur on *I*_Kr__–__tail_. **(E)** The comparison of average *I*_Kr__–__tail_ percentage values of three groups (*n* = 8, ***P < 0.001 vs. control, ^&&&^*P* < 0.001 vs. 10 μmol/L Cur).

The *I*–*V* and concentration–response relationships of Cur on *I*_Kr_ was evaluated by measuring the tail currents (*I*_K__–__tail_) (Figure 4A). The *I*–*V* relationships curves showed that Cur inhibited *I*_K__–__tail_ in a concentration-dependent manner (Figure 4B). The concentration–response relationship curves were fitted with a Hill function and the value of IC_50_ was calculated to be 9.96 μmol/L (Figure 4C). The *I*_K__–__tail_ was inhibited by 10 μmol/L Cur and could be reversed by washing out (Figures 4D,E).

### Effects of Cur on the Early Afterdepolarization (EAD)-Like and Delayed Afterdepolarization (DAD)-Like Arrhythmogenic Activity in Ventricular Myocytes

**FIGURE 5:**
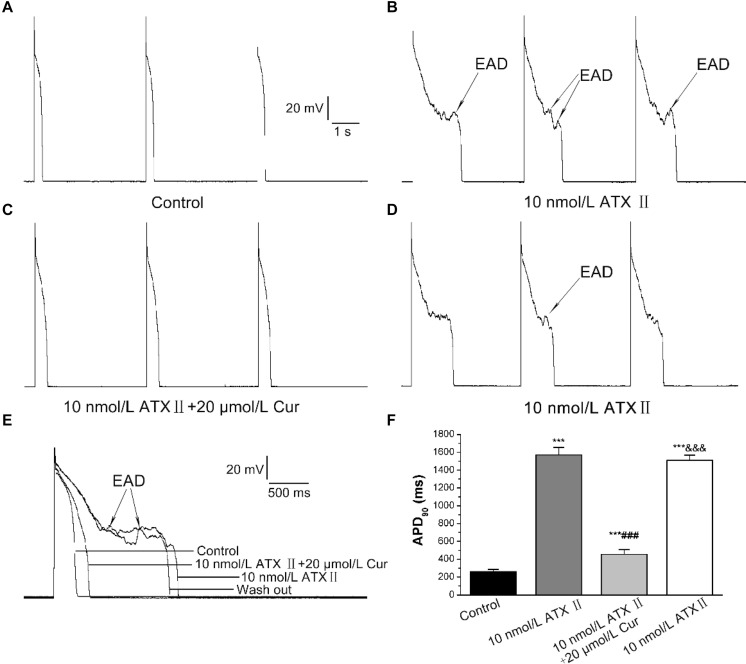
The antagonistic effects of Cur on the prolonged APD and early afterdepolarization (EAD) in ventricular myocytes. **(A–D)** Example records of APs with a train-of-three stimulation at a lower frequency of 0.25 Hz for the control group, ATX II group, Cur-treated group, and wash out group, respectively. **(E)** Example records of single APs at a frequency of 0.25 Hz on the different groups. **(F)** The comparison of average APD_90_ values of four groups (*n* = 11, ***P < 0.001 vs. control; ^###^*P* < 0.001 vs. 10 nmol/L ATX II; ^&&&^*P* < 0.001 vs. 10 μmol/L ATX II with 20 μmol/L Cur).

*I*_Na.L_ opener-ATX II was used to induce a prolongation of APD and the occurrence of EADs to explore the potential antagonistic effects of Cur on EADs. The results showed that 10 nmol/L ATX II effectively prolonged APD (*n* = 11, Figure 5A), and all 11 ventricular myocytes occurred EADs (Figure 5B). For given Cur at a concentration of 20 μmol/L, the prolonged APD was significantly shortened and the occurrence of EADs was completely eliminated (11/11, Figure 5C). Moreover, EADs reappeared and APD prolonged again after continuous washing with external solution containing 10 nmol/L ATX II (11/11, Figures 5D–F).

**FIGURE 6:**
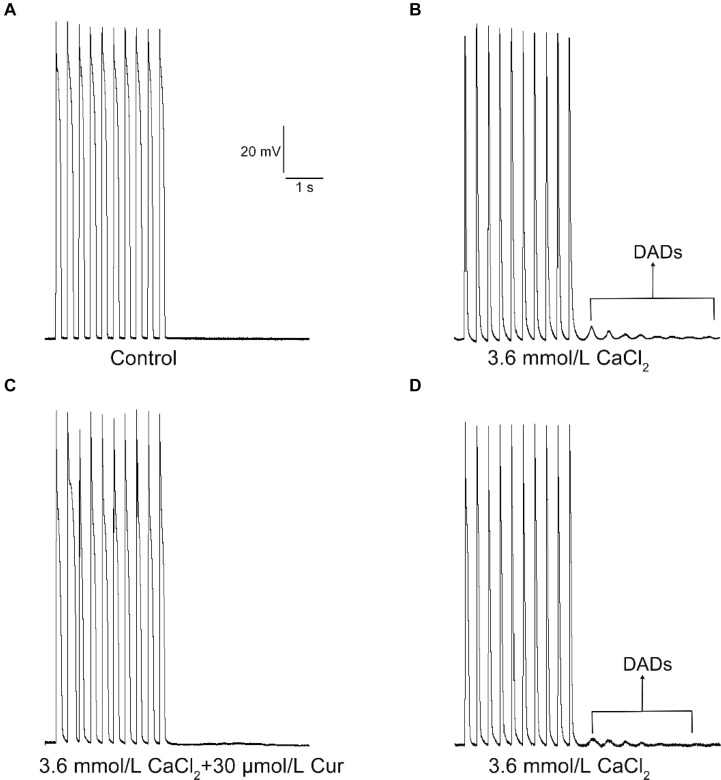
The effects of Cur on delayed afterdepolarization (DAD) in ventricular myocytes. **(A–D)** Example records of APs with a train-of-10 stimulation at a higher frequency of 5 Hz, and the cycle length was 7 s. **(A–D)** Represent the control group, DAD group, Cur-treated group, and wash out group, respectively.

In the present study, the DADs were elicited by increasing the concentration of CaCl_2_ in the external solution to 3.6 mmol/L. DADs occurred in 9 of 9 ventricular myocytes (Figures 6A,B). At a concentration of 30 μmol/L, Cur completely suppressed the DADs in 9 of 9 ventricular myocytes (9/9, Figure 6C). The DADs reappeared after washing with the external solution containing 3.6 mmol/L CaCl_2_ (9/9, Figure 6D).

### The Antiarrhythmic Effects of Cur on Langendorff-Perfused Rabbit Hearts Suffered From I/R Injury

**FIGURE 7:**
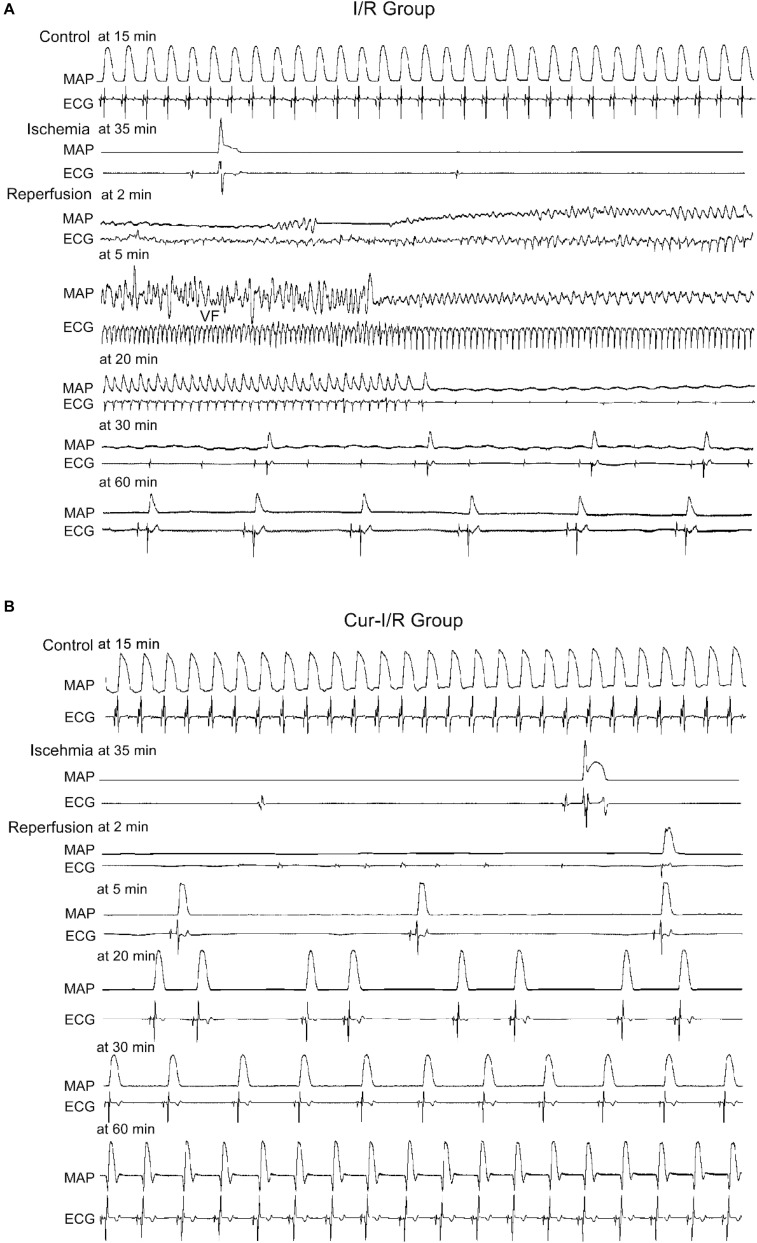
The effects of Cur on isolated rabbit hearts that suffered from ischemia–reperfusion (I/R). **(A,B)** Example records of MAP and ECG of the Langendorff-perfused hearts in the I/R group **(A)** and Cur-I/R group **(B)**.

**FIGURE 8:**
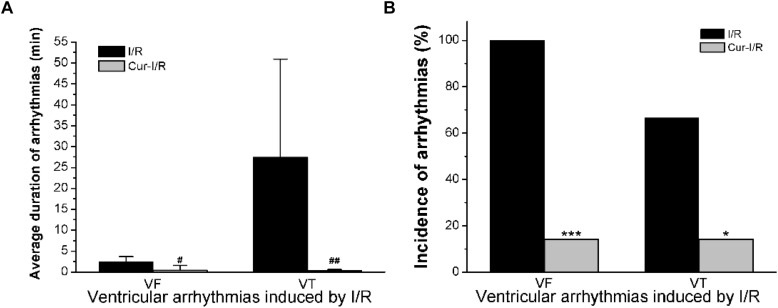
The histogram of I/R experiments results. **(A)** The comparison of average duration of ventricular fibrillation (VF) and ventricular tachycardia (VT) after reperfusion in the two groups (^#^*P* < 0.05, ^##^*P* < 0.01 vs. I/R group). **(B)** The comparison of the incidence of VF and VT in the two groups (*P < 0.05, ***P < 0.001 vs. I/R group).

In the I/R group, the results showed that in 8 of 12 cases, both ventricular fibrillation (VF) and ventricular tachycardia (VT) occurred, while in the other 4 cases, only VF occurred (Figure 7A). In the Cur-I/R group (Figure 7B), there was a pretreatment of 30 μmol/L Cur before ischemia. VF and VT only occurred in one of seven I/R injury hearts; nevertheless, no VF or VT occurred in the other six cases and sinus rhythm was restored after reperfusion. The effect of Cur on I/R-induced arrhythmias was evaluated by comparing the average duration and incidence of VF and VT between two groups. The results showed that Cur significantly reduced the incidence and average duration of the VT and VF after reperfusion (Figures 8A,B). It can be seen that Cur revealed an anti-arrhythmic effect on isolated rabbit hearts subjected to I/R.

## Discussion

Cur is a kind of active alkali extracted from turmeric rhizomes and has long been used as a traditional medicine, especially the spice and coloring agent in food (Kumar et al., 2017). Over the past half century, extensive researches on Cur have shown that it is a highly pleiotropic molecule that has multiple pharmacological activities (Burashnikov and Antzelevitch, 2009; Gupta et al., 2013). In this study, we investigated the influence of Cur on the electrophysiology of rabbit ventricular myocytes and explored its antiarrhythmic property. The results of the present study suggested that Cur is a multi-ion channel blocker, which inhibited *I*_Ca.L_, *I*_Na.L_, *I*_Na.T_, *I*_Kr_ and preferentially blocks *I*_Na.L_, cumulatively resulting in the changes of AP waveform. Additionally, Cur exhibited an antagonism on EAD and DAD-like arrhythmias in ventricular myocytes, and also reduced the occurrence of VF and VT in Langendorff-perfused rabbit hearts subjected to I/R.

To our knowledge, the effect of Cur on the AP of ventricular myocytes is not clear. The generation of APs is the response to the organized activities of several transmembrane ionic currents in cardiomyocytes, and the AP waveform will be remodeled by disrupting the activity of these ionic currents. *I*_Na.T_ is the fundamental inward ion current that initiates the depolarization of the AP in ventricular myocytes (Rashid and Kuyucak, 2018). Our results found that 30 μmol/L Cur has no significant effect on APA or *V*_max_, which is consistent with the findings that 30 μmol/L Cur has no significant inhibitory effect on *I*_Na.T_, suggesting that Cur did not affect the generation or conduction of AP in ventricular myocytes. It is well known that the APD related to the balance between inward Ca^2+^ and outward K^+^ channel. Moreover, latest research has confirmed that the basal endogenous *I*_Na.L_ is also a significant contributor to APD (Song and Belardinelli, 2017). In our study, Cur shortened APD_50_ more significantly than APD_90_ (APD_50_ and APD_90_ was shortened by 17 and 7%) which may be related to the blocking effects of Cur on *I*_Na.L_, *I*_Ca.L_, and *I*_Kr_. The suppression of *I*_Kr_ would have caused the retardation and prolongation of APD, but the inhibitory effects of Cur on *I*_Ca.L_ and *I*_Na.L_ make a contrary effect on the prolongation of APD. The slight shortening of APD_90_ (only shortened by 7%) may not show up on the ECG, which was consistent with the previous study where Cur (1 mg/ml) had no significant influence on QT interval *in vivo* (Hu et al., 2012).

As we all know, the *I*_Ca.L_-triggered excitation–contraction coupling is essential for cardio function and plays a crucial role in cardiac arrhythmias (Guia et al., 2001). The aberrant activation of *I*_Ca.L_ will cause intracellular Ca^2+^ concentration ([Ca^2+^]i) overload and lead to disequilibrium of [Ca^2+^]i homeostasis (Fabiato, 1983; Inui and Fleischer, 1988; Meissner et al., 1988; Cannell et al., 2013). In the present study, we found that Cur not only reduced the *I*_Ca.L_ but also played a role in the kinetics of L-type calcium channels, which accelerated steady-state inactivation and prolonged time-dependent recovery from inactivation. These various regulatory effects of Cur on *I*_Ca.L_ synergistically reduced the [Ca^2+^]i. The inhibition of *I*_Ca.L_ may prevent the [Ca^2+^]i overload and thus play an antiarrhythmic role (Damasceno et al., 2012).

In many pathological conditions (e.g., oxidative stress, ischemia, and heart failure), the augmentation of *I*_Na.L_ will prolong the APD and facilitate EADs (Tang et al., 2012; Toischer et al., 2013). Moreover, the increased *I*_Na.L_ may cause intracellular Na^+^ ([Na^+^]i) overload, which is followed by an increased influx of Ca^2+^ via enhanced reverse Na^+^/Ca^2+^ exchanger (*I*_NCX_), resulting in [Na^+^]i-dependent [Ca^2+^]i overload. This failure to maintain the homeostasis of [Na^+^]i and [Ca^2+^]i will induce arrhythmias. Thus, effective inhibition of *I*_Na.L_ can reduce [Na^+^]i-dependent [Ca^2+^]i overload and may suppress its induced arrhythmias (Cao et al., 2018; Li et al., 2013; Yang et al., 2011). At present, the traditional class I C Na^+^ channel inhibitors are potent inhibitors of *I*_Na.L_ used as AADs, but safety concerns have limited the use of these agents in clinical practice due to the significant inhibition of *I*_Na.T_, such as Mexiletine, Quinidine, and Flecainide with an IC_50_ ratio on *I*_Na.T_ and *I*_Na.L_ of 1.96, 1.1, and 1, respectively, and clinically used AADs, such as Disopyramide and Procaine, which have a smaller IC_50_ ratio on *I*_Na.T_ and *I*_Na.L_ (Guo and Jenkinson, 2018). This non-negligible inhibition on *I*_Na.T_ may slow the conduction of AP especially in hearts with structural disease, leading to cardiac arrhythmia. Therefore, there is an urgent need to explore a potent inhibitor of *I*_Na.L_ without inhibiting *I*_Na.T_ obviously. In our research, we found that the inhibitory effects of Cur on *I*_Na.L_ are far greater than *I*_Na.T_ with an IC_50_ ratio on *I*_Na.T_ and *I*_Na.L_ of 52.97. Even at a concentration of about 3.98-fold, the IC_50_ value of *I*_Na.L_, Cur only reduced *I*_Na.T_ by 4%. Ranolazine appears to be the most clinically selective inhibitor of late *I*_Na.L_ with an IC_50_ ratio on *I*_Na.T_ and *I*_Na.L_ of 4.49 (Guo and Jenkinson, 2018). Similar to Cur, ranolazine not only inhibits *I*_Na.L_ and *I*_Na.T_ but also blocks *I*_Kr_. It is a pity that there is no *I*_Na.L_ inhibitor that is more effective and selective than ranolazine, which remains the preferred *I*_Na.L_ inhibitor (Antzelevitch et al., 2011). In this sense, it may be safer for Cur to reduce *I*_Na.L_ instead of *I*_Na.T_ than the abovementioned class I C Na^+^ channel inhibitors.

From the above discussion, Cur was a multi-ion channel blocker that preferentially blocks *I*_Na.L_, which had the potential to prevent [Ca^2+^]i overload by inhibiting *I*_Ca.L_ and *I*_Na.L_. Previous clinical and experimental studies have indicated that the traditional natural products with multi-ion channel blocking effects not only inhibited the occurrence of arrhythmias to some extent but also have lower toxicity and side effect (Brenyo and Aktas, 2014; Chuang et al., 2016; Wang et al., 2016). Moreover, multi-ion channel blockers can decrease the risk of pro-arrhythmia, which is an obstacle of the traditional AADs (Nattel and Carlsson, 2006). Cur, a traditional natural product with a multi-ion channel blocker that preferentially blocks *I*_Na.L_, may have lower toxicity and fewer side effects, with the addition of various clinical trials showing that Cur does not cause any adverse complications on liver and kidney function and it is safe even at high dose (Rahmani et al., 2018), which makes it worthy for further studies as a candidate of AAD.

Researches have shown that increased *I*_Na.L_ and *I*_Ca.L_ will not only prolong APD and cause EADs but also lead to DADs and triggered activities (Wagner et al., 2011). The EADs may contribute to the occurrence of ventricular premature contraction and torsade de pointes, thus leading to VF (Binah and Rosen, 1992). We found that Cur not only significantly shortened ATX II-prolonged APD but also suppressed the occurrence of EADs. In this study, the antagonistic effects of Cur on EADs may be related to its inhibitory effects on ATX II-increased *I*_Na.L_, which had been investigated in the present study. In addition to EADs, the DADs were also implicated in arrhythmias and SCDs. DADs are the membrane potential oscillation that occurs after cardiomyocytes are completely repolarized. This membrane potential oscillation was caused by the increase of [Ca^2+^]i, leading to the enhanced *I*_NCX_, which in turn generated the transient inward currents (Ter Keurs and Boyden, 2007). Therefore, it can be concluded that the DADs can be suppressed by reducing the [Ca^2+^]i. In this investigation, Cur displayed a significant antagonistic effect on the DAD-like arrhythmogenic activity in ventricular myocytes, which may be related to the inhibitory effects of Cur on *I*_Ca.L_. Thus, it seems that Cur has the effect of inhibiting EAD-like and DAD-like arrhythmogenic activity at the cellular level.

Coronary disease is one of the leading causes of death and disability of CVDs, and the coronary recanalization usually cannot prevent cardiomyocyte death, but instead facilitate severe arrhythmias after reperfusion (Ruiz-Meana and Garcia-Dorado, 2009). Previous studies have demonstrated that I/R injury-induced arrhythmias related to [Ca^2+^]i overload, which is mainly caused by the significant augmentation of *I*_Na.L_ (Soliman et al., 2012). During the ischemia stage, the increased intracellular H_2_O_2_ and acidosis lead to the increase of inward Na^+^ currents, which causes intracellular Ca^2+^ aggregation via the reverse *I*_NCX_. *I*_Na.L_ is intensified to rectify acidosis after reperfusion, which will lead to more serious destruction of intracellular Ca^2+^ homeostasis, leading to severe arrhythmia (Ruiz-Meana and Garcia-Dorado, 2009). Thus, alleviating the [Ca^2+^]i overload will contribute to the suppression of I/R-induced arrhythmias. In the present study, Cur exhibited an inhibitory role on *I*_Ca.L_ and *I*_Na.L_, which means that Cur can reduce [Ca^2+^]i. The findings also demonstrated that Cur can prevent the occurrence of arrhythmias after reperfusion, which is beneficial for the recovery of isolated heart suffering from I/R injury. The suppression effects of Cur on I/R-induced arrhythmias may be related to its inhibition on *I*_Ca.L_ and *I*_Na.L_. The results of the study show that Cur has a certain antagonized effect on cardiac arrhythmia and has potential application prospects for AADs.

## Conclusion

In conclusion, Cur is a multi-ion channel blocker that inhibits *I*_Ca.L_ and *I*_Kr_ and preferentially blocks *I*_Na.L_, shortens APD, suppresses EADs and DADs at the cellular level, and prevents I/R-induced arrhythmia at the organ level.

## Data Availability Statement

The original contributions presented in the study are included in the article/Supplementary Material, further inquiries can be directed to the corresponding author.

## Ethics Statement

The animal study was reviewed and approved by the Institutional Animal Care and Use of Wuhan University of Science and Technology.

## Author Contributions

JM managed the experimental design and thesis supervision. LS managed the experiments, adjustment of experimental scheme, data analysis, creating the figure, and manuscript preparation. ZZ managed preliminary experiments, data collection and analysis, and involved in the manuscript preparation. LH managed the experiment in organ level and collected data based on the result. P-HZ managed the preparation of experimental materials and experimental method guidance in the whole study. P-PZ assisted in material preparation. ZC assisted in creating the figures and the manuscript refinement. ZL assisted in solution preparation and improving the experiment method. All authors contributed to the article and approved the submitted version.

## Conflict of Interest

The authors declare that the research was conducted in the absence of any commercial or financial relationships that could be construed as a potential conflict of interest.

## Abbreviations

AADs: Antiarrhythmic drugs
APA: Action potential amplitude
APD_50_ and APD_90_: 50% and 90% repolarization of action potential
Cur: Curcumin
CVDs: Cardiovascular diseases
DAD: proarrhythmic Delayed afterdepolarization
EAD: Early afterdepolarization
ECG: Electrocardiogram
*I*_Ca.L_: L-type calcium current
*I*_Na.T_: Transient-sodium current
*I*_Na.L_: Late-sodium current
*I*_Kr_: Rapidly delayed rectifying potassium current
*I*_NCX_: Na^+^/Ca^2+^ exchanger
I/R: Ischemia–reperfusion
MAP: Monophasic action potential
RMP: Resting membrane potential
*V*_max_: Maximum depolarization velocity
VF: Ventricular fibrillation
VT: Ventricular tachycardia
[Ca^2+^]i: Intracellular Ca^2+^ concentration
[Na^+^]i: Intracellular Na^+^ concentration.
