# Supplementary material for: Curcumin, a Multi-Ion Channel Blocker That Preferentially Blocks Late Na+ Current and Prevents I/R-Induced Arrhythmias
Source: Front Physiol. 2020 Aug 21;11:978. doi: 10.3389/fphys.2020.00978 (PMC7472421; doi:10.3389/fphys.2020.00978)
Supplement: Supplementary file 1 [file Data_Sheet_1.zip › 8Figure+1table/Table1.docx]

**Table1. Effects of Cur on the parameters of APs in rabbit ventricular myocytes**

| Parameters | Control | 30 μmol/L Cur | Wash out |
| --- | --- | --- | --- |
| RMP (mV) | -77±3 | -75±4 | -74±5 |
| APA (mV) | 123±7 | 114±9 | 114±10 |
| APD50 (ms) | 239±15 | 199±22^**^ | 226±21^§^ |
| APD90 (ms) | 271±8 | 252±11^*^ | 282±29^§§^ |
| Vmax (V/s) | 186±12 | 164±25 | 159±18 |

**RMP:** Resting membrane potential; **APA:** action potential amplitude; **APD_50_:** 50% repolarization of AP; **APD_90_:** 90% repolarization of AP; **Vmax:** maximum depolarization velocity. (n=12, ^*^*P*<0.05, ^**^*P*<0.01 vs. control; ^§^*P*<0.05, ^§§^*P*<0.01 vs. 30μmol/L Cur).
