# Supplementary figures and images for: Curcumin, a Multi-Ion Channel Blocker That Preferentially Blocks Late Na+ Current and Prevents I/R-Induced Arrhythmias
Source: Front Physiol. 2020 Aug 21;11:978. doi: 10.3389/fphys.2020.00978 (PMC7472421; doi:10.3389/fphys.2020.00978)

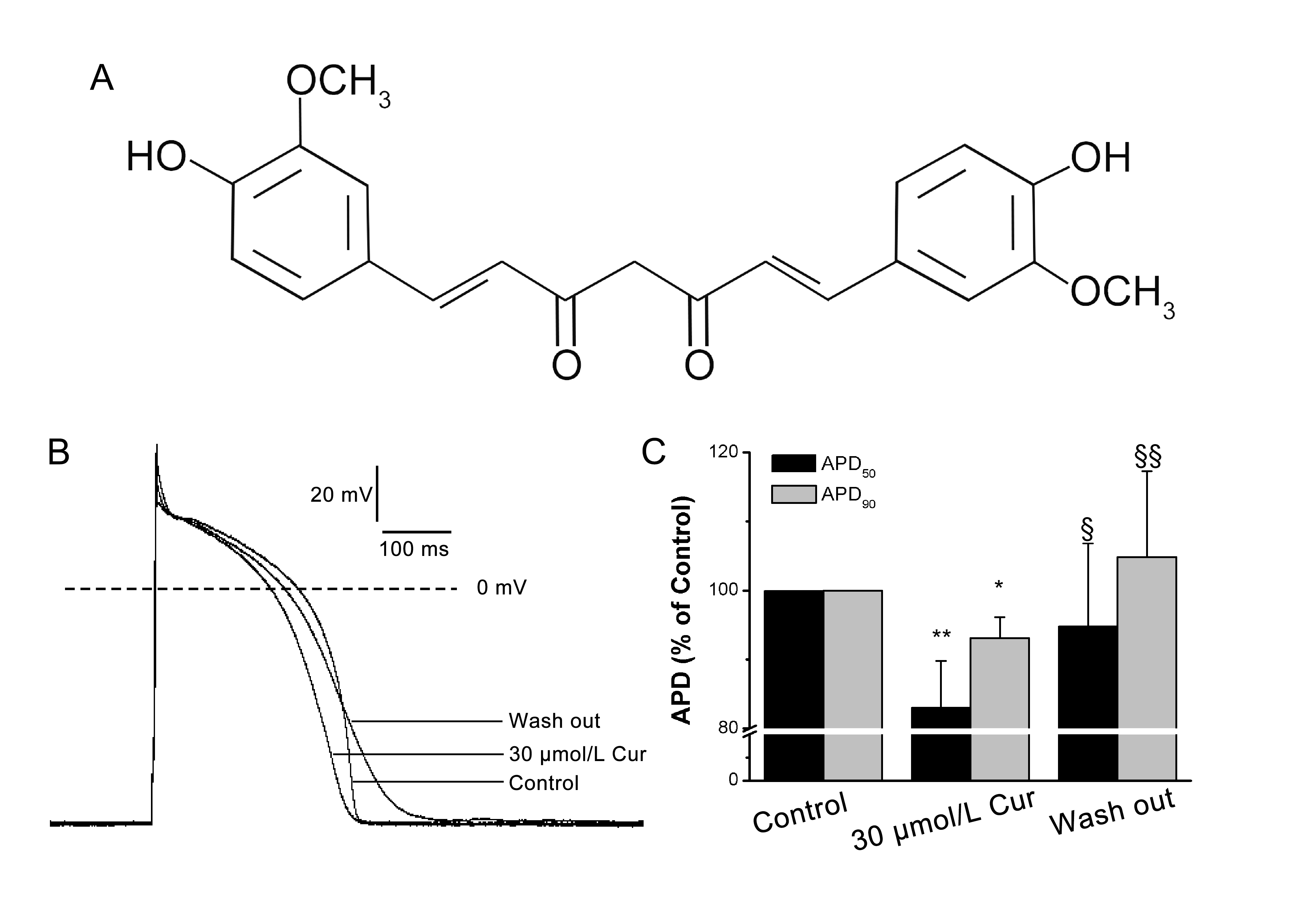

Supplement: Supplementary file 1 [file Data_Sheet_1.zip › 8Figure+1table/FIG-1.tif]

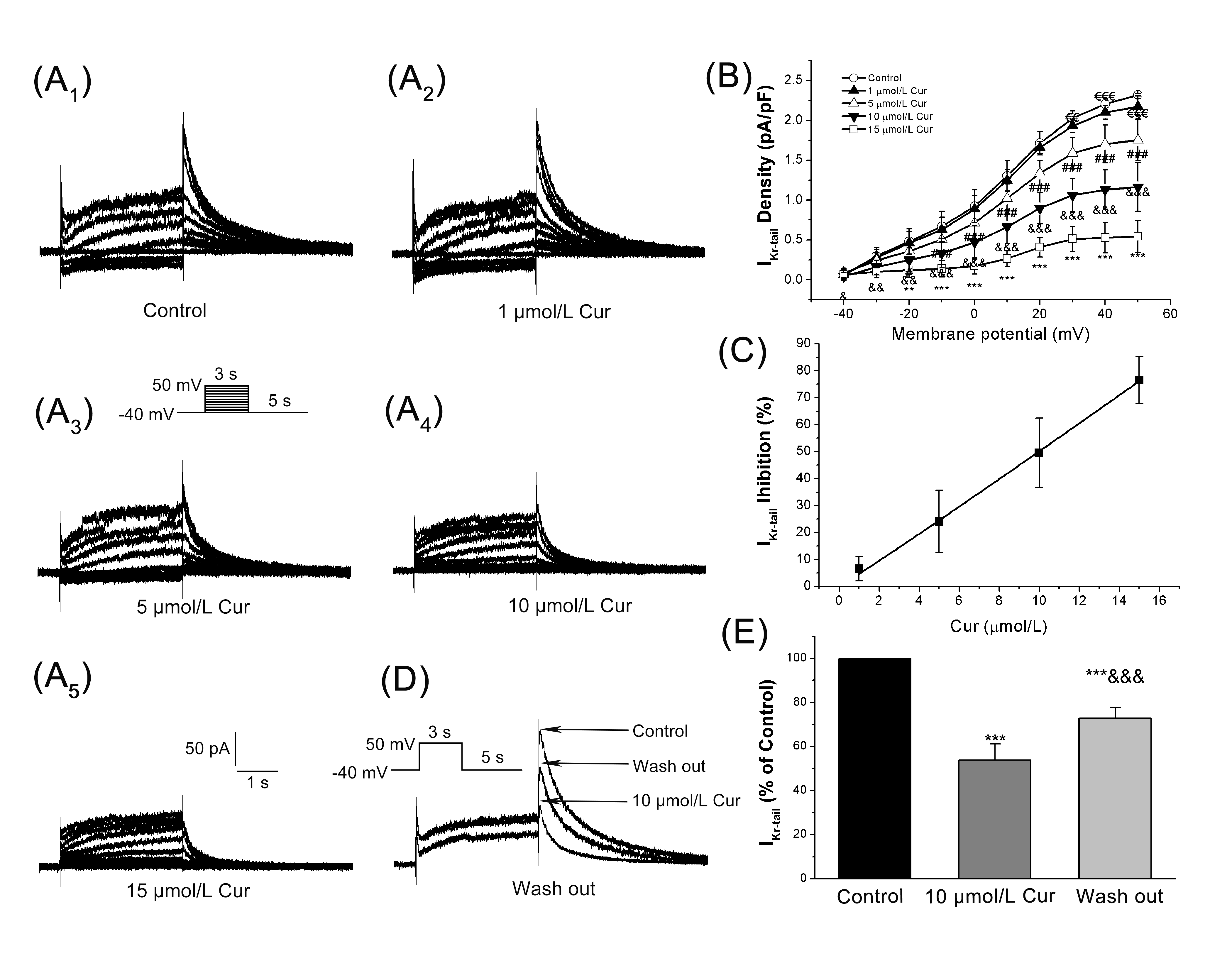

Supplement: Supplementary file 1 [file Data_Sheet_1.zip › 8Figure+1table/Figure-4.tif]

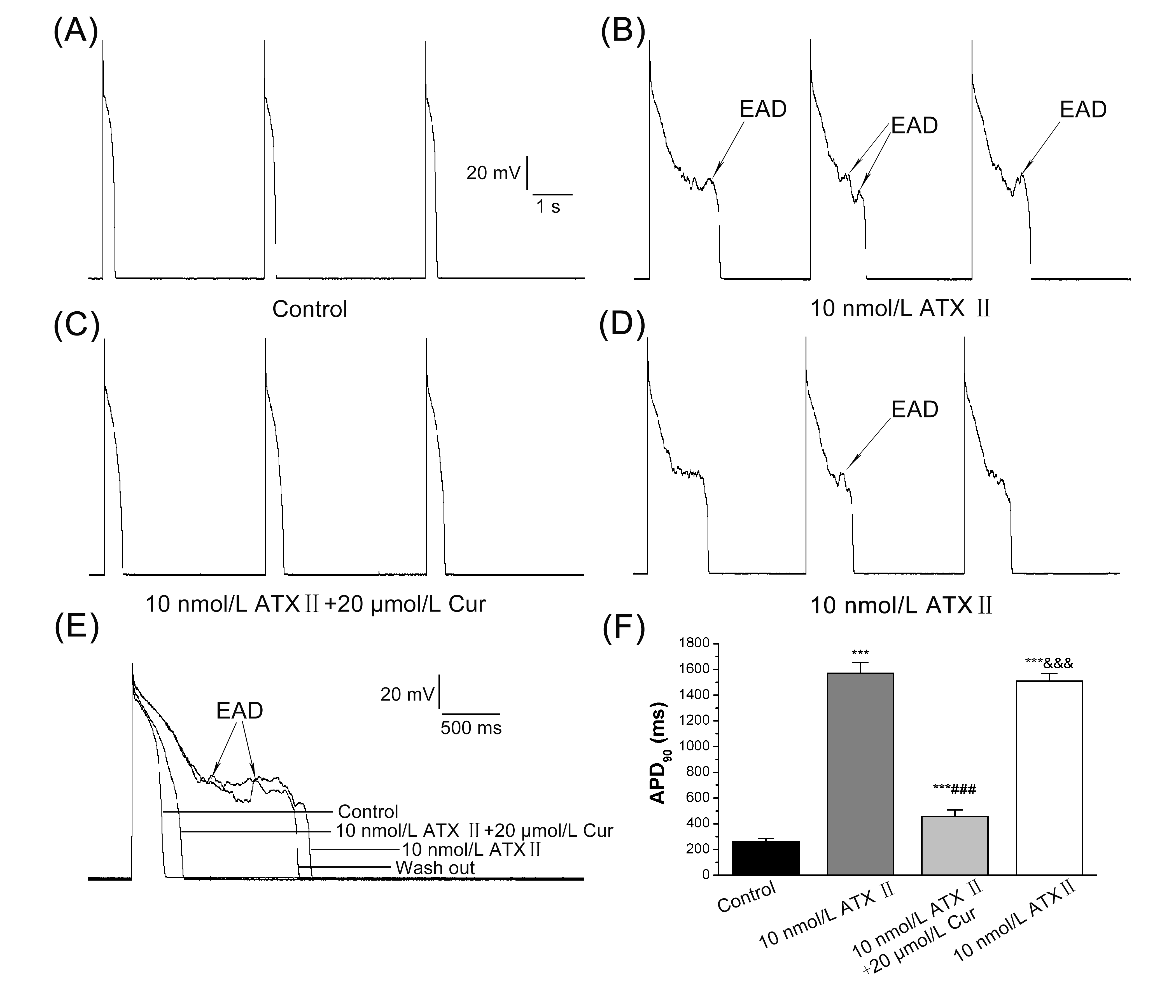

Supplement: Supplementary file 1 [file Data_Sheet_1.zip › 8Figure+1table/Figure-5.tif]

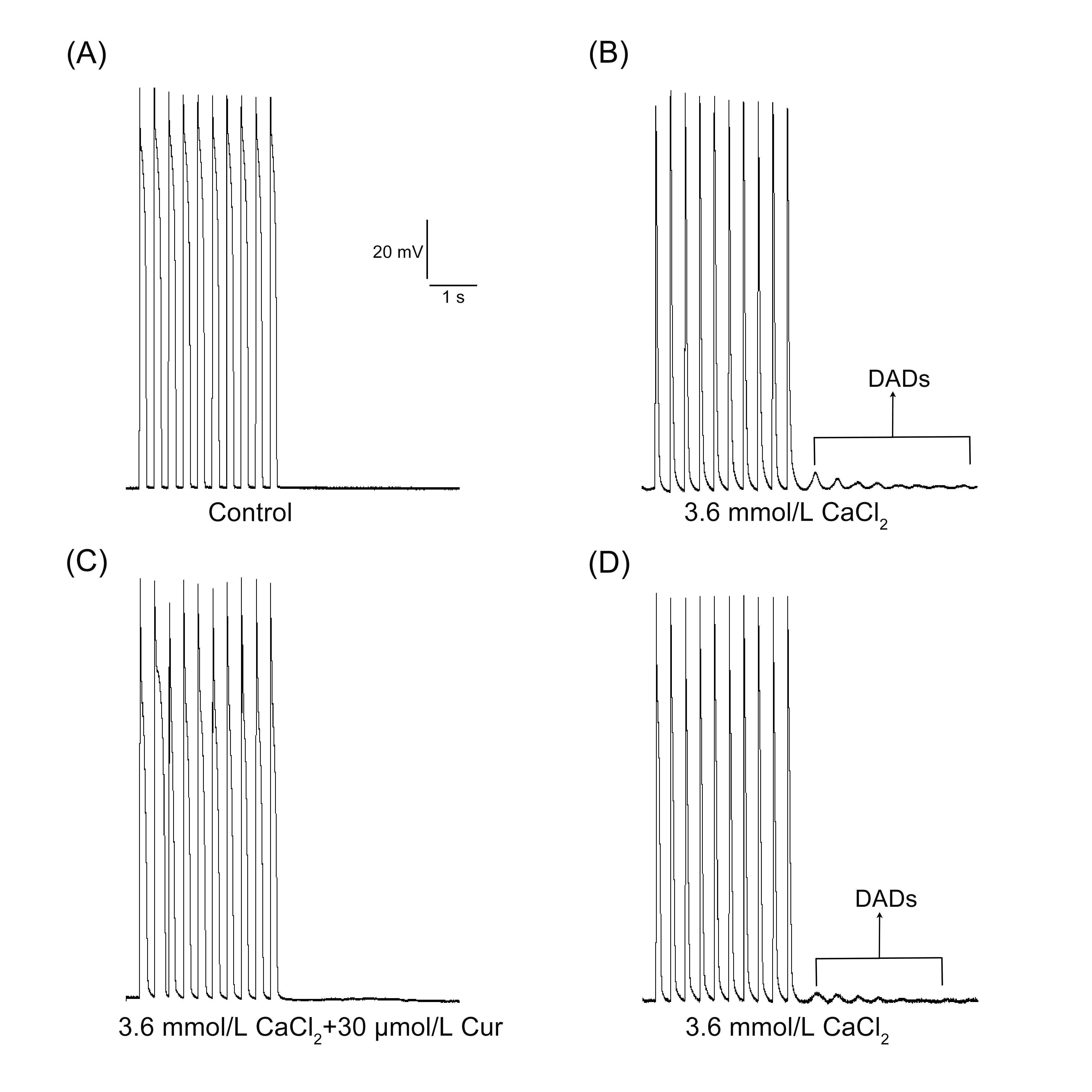

Supplement: Supplementary file 1 [file Data_Sheet_1.zip › 8Figure+1table/Figure-6.tif]

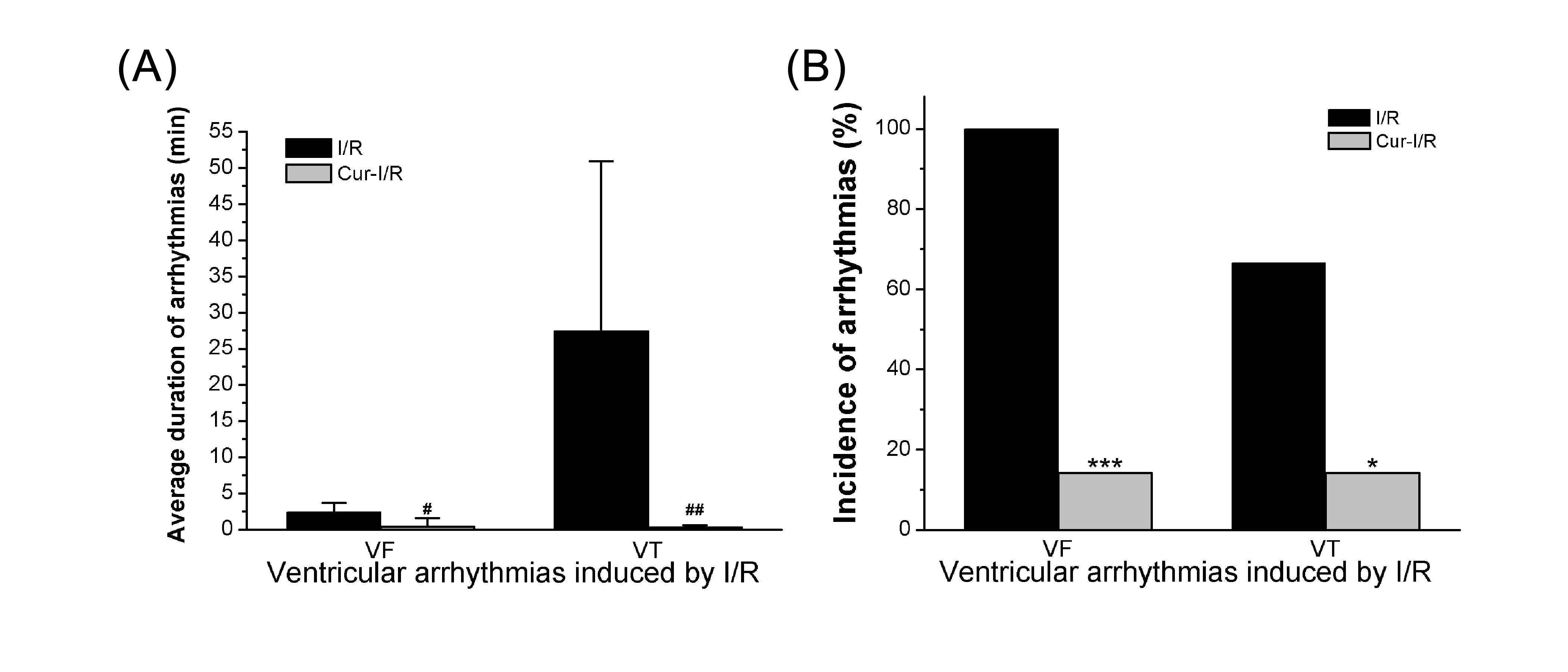

Supplement: Supplementary file 1 [file Data_Sheet_1.zip › 8Figure+1table/Figure-8.tif]
